# Epigenetic modification of PHLDA2 is associated with tumor microenvironment and unfavorable outcome of immune checkpoint inhibitor-based therapies in clear cell renal cell carcinoma

**DOI:** 10.1186/s40001-024-01939-9

**Published:** 2024-07-20

**Authors:** Junjie Zhao, Xiuyi Pan, Zilin Wang, Yuntian Chen, Dingbang Liu, Yu Shen, Xinyuan Wei, Chenhao Xu, Xingming Zhang, Xu Hu, Junru Chen, Jinge Zhao, Bo Tang, Guangxi Sun, Pengfei Shen, Zhenhua Liu, Hao Zeng, Jiayu Liang

**Affiliations:** 1 Department of Urology, Institute of Urology, West China Hospital, Sichuan University, No. 37 Guoxue Alley, Wuhou District, Chengdu, Sichuan China; 2 Department of Pathology, West China Hospital, Sichuan University, Chengdu, China; 3 Department of Radiology, West China Hospital, Sichuan University, Chengdu, China

**Keywords:** PHLDA2, Clear cell renal cell carcinoma, DNA hypomethylation, Immune microenvironment, Immune checkpoint inhibitor, Biomarker

## Abstract

**Background:**

A substantial proportion of patients with metastatic clear cell renal cell carcinoma (ccRCC) cannot derive benefit from immune checkpoint inhibitor (ICI) plus anti-angiogenic agent combination therapy, making identification of predictive biomarkers an urgent need. The members of pleckstrin homology-like domain family A (PHLDA) play critical roles in multiple cancers, whereas their roles in ccRCC remain unknown.

**Methods:**

Transcriptomic, clinical, genetic alteration and DNA methylation data were obtained for integrated analyses from TCGA database. RNA sequencing was performed on 117 primary tumors and 79 normal kidney tissues from our center. Gene Ontology and Kyoto Encyclopedia of Genes and Genomes analysis, gene set enrichment analysis were performed to explore transcriptomic features. Data from three randomized controlled trials (RCT), including CheckMate025, IMmotion151, JAVELIN101, were obtained for validation.

**Results:**

Members of PHLDA family were dysregulated in pan-cancer. Elevated PHLDA2 expression was associated with adverse clinicopathologic parameters and worse prognosis in ccRCC. Aberrant DNA hypomethylation contributed to up-regulation of PHLDA2. An immunosuppressive microenvironment featured by high infiltrates of Tregs and cancer-associated fibroblasts, was observed in ccRCC with higher PHLDA2 expression. Utilizing data from three RCTs, the association of elevated PHLDA2 expression with poor therapeutic efficacy of ICI plus anti-angiogenic combination therapy was confirmed.

**Conclusions:**

Our study revealed that elevated PHLDA2 expression regulated by DNA hypomethylation was correlated with poor prognosis and immunosuppressive microenvironment, and highlighted the role of PHLDA2 as a robust biomarker for predicting therapeutic efficacy of ICI plus anti-angiogenic agent combination therapy in ccRCC, which expand the dimension of precision medicine.

**Supplementary Information:**

The online version contains supplementary material available at 10.1186/s40001-024-01939-9.

## Introduction

Clear cell renal cell carcinoma (ccRCC), the most prevalent histologic subtype of renal cell carcinoma (RCC) with heterogeneous prognosis, accounts for about 70–80% of newly diagnosed RCC cases [1]. For localized disease, partial or radical nephrectomy remains the major curable management, whereas approximately 30% localized RCC patients will unavoidably develop recurrence or metastasis after tumor resection, thus requiring systemic treatment ultimately [2]. Over the past two decades, given that aberrant activation of angiogenesis produces a marked effect in ccRCC pathogenesis, anti-angiogenic agents entered the clinic and became the standard first-line treatment options [3]. More recently, the approval of immune checkpoint inhibitors (ICI) and immunotherapy-based combination strategies, especially ICI plus anti-angiogenic agent, has revolutionarily expanded the treatment landscape for the metastatic settings, with much more favorable therapeutic response [4–8]. However, due to the high heterogeneity of ccRCC, a substantial proportion of patients cannot derive benefit from ICI plus anti-angiogenic agent combination therapy [9]. Therefore, identification of reliable biomarkers for responses to ICI plus anti-angiogenic agent combination therapy is urgently needed to optimize therapeutic decision-making in ccRCC.

The pleckstrin homology-like domain (PHLD) proteins are multifunctional proteins involved in normal metabolic homeostasis and human pathology [10]. The PHLD class of proteins is arranged in two separate families, PHLDA and PHLDB. So far, PHLDA family members, including PHLDA1, PHLDA2, PHLDA3, have gained more attention due to their association with various cancers. With regard to PHLDA1, the oncogenic role of PHLDA1 was controversial among different malignancies. Within glioblastoma, ovarian cancer, and colon cancer, PHLDA1 has been identified as an oncogene, involving in tumor cell proliferation, anti-apoptosis, and migration [11–13]. Nevertheless, in breast cancer, PHLDA1 could negatively regulate Aurora A and antagonize Aurora A-mediated oncogenic pathways, and decreased PHLDA1 expression was associated with poorer prognosis [14, 15]. Remarkably, knockdown of PHLDA1 was sufficient to confer de-novo resistance to tyrosine kinase receptor (TKI) targeted therapy [16]. For PHLDA2, up-regulation of PHLDA2 could lead to proliferation of tumor cells in liver cancer, glioma, and colorectal cancer [17–19]. Liu et al. further revealed that PHLA2 promoted 5-Fu resistance, suggesting that PHLDA2 might be an effective target in colon cancer to overcome resistance for chemotherapy [20]. However, PHLDA2 could also suppress tumor development by triggering a distinct ferroptosis response, suggesting complex roles of PHLDA2 in tumorigenesis [21]. PHLDA3 is a p53-regulated Akt repressor which inhibits Akt translocation to the cell membrane and activation [22]. Down-regulation of PHLDA3 has been reported in lung cancer and pancreatic neuroendocrine tumors, whereas Lei et al. found that PHLDA3 could activate the Wnt signaling pathway to promote the proliferation and invasion of lung cancer cells [22–24]. Taken together, the PHLDA family members can both promote and suppress tumor progression, which depend on the specific type of cancer. Unfortunately, the role of PHLDA family members in pathogenesis of ccRCC have not been well understood, which arises our research interest.

To address the unmet needs mentioned above, we aimed to explore the potential value of the PHLDA family members in the diagnosis and prognosis of ccRCC using multi-omics data. Furthermore, clinical and transcriptomic data of three randomized controlled trials (RCT), including CheckMate025, IMmotion151 and JAVELIN101, were utilized to evaluate the association of the expression of PHLDA family members with treatment efficacy [5–7]. Our results suggested that elevated PHLDA2 expression could be served as an independent high-risk prognostic factor, predicting worse therapeutic efficacy of ICI plus anti-angiogenic agent combination therapy in ccRCC.

## Materials and methods

### Gene expression analysis

The RNA-sequencing (RNA-seq) data of 33 types of tumors and adjacent normal tissues, along with clinical information for ccRCC patients, were derived from the TCGA database. Patients who had missing data were excluded from the study. In parallel, transcriptomic data of ccRCC tumors and normal kidney tissues in two GEO data sets, including GSE40435, GSE53757, were obtained. RNA-seq was performed on primary tumors and adjacent normal tissues from patients with ccRCC who underwent surgery in West China Hospital, Sichuan University. The study protocol was approved by the Ethics Committee of West China Hospital, Sichuan University. The study was conducted in accordance with the Declaration of Helsinki, and written informed consent was obtained from each patient. Wilcoxon rank-sum test was applied to compare the expression level of PHLDA1, PHLDA2, and PHLDA3 between tumor and normal tissues. The “ggplot2” R package was utilized to visualize the results.

### RNA extraction, library preparation, and sequencing

Primary tumors and adjacent normal tissues from patients with ccRCC who performed operations in West China Hospital, Sichuan University were collected, frozen in liquid nitrogen. Total RNA was extracted using TRIzol Reagent (Invitrogen, cat. NO 15596026) following the methods by Chomczynski et al. [25]. After RNA extraction, DNA digestion was carried out by DNaseI. RNA quality was determined by examining A260/A280 with Nanodrop™ OneCspectrophotometer (Thermo Fisher Scientific Inc). RNA Integrity was confirmed by 1.5% agarose gel electrophoresis. Qualified RNAs were finally quantified by Qubit3.0 with QubitTM RNA Broad Range Assay kit (Life Technologies, Q10210).

According to the manufacturer’s instruction, 2 μg total RNAs were used for stranded RNA sequencing library preparation using KC-Digital™ Stranded mRNA Library Prep Kit for Illumina® (Catalog NO. DR08502, Wuhan Seqhealth Co., Ltd. China). Using unique molecular identifier of eight random bases to label the pre-amplified cDNA molecules, the kit eliminates duplication bias in polymerase chain reaction and sequencing steps. The library products were enriched corresponding to 200–500 bps, quantified and sequenced on DNBSEQ-T7 sequencer (MGI Tech Co., Ltd. China) with PE150 model.

### Clinicopathologic parameters, and prognosis analysis

Clinical information of ccRCC patients, including age, gender, pT stage, pN stage, metastatic status, ISUP grade, was collected from the TCGA database. The correlation between transcriptomic data and clinicopathologic parameters was analyzed using Wilcoxon rank-sum test and was visualized using the “ggplot2” R package. The ClearCode34-based model was used for the stratification of ccRCC samples into ccA and ccB subtypes [26].

Kaplan–Meier (KM) survival analysis was performed for overall survival (OS), disease-specific survival (DSS), and progression-free interval (PFI). Log-rank test was used to determine the significance of difference in prognosis. The results were visualized by “survminer” and “ggplot2” R packages. Univariate and multivariate Cox regression analyses were performed to investigate whether expression level of PHLDA family members could be served as an independent prognostic factor of ccRCC patients. Parameters with a *p* < 0.1 in univariate Cox regression analysis were included into multivariate Cox regression analysis.

### Genetic alteration and DNA methylation analysis

Information of genetic alterations were obtained from the TCGA database, including somatic mutations and copy number variations (CNV). The association of PHLDA2 expression and CNV was analyzed using Wilcoxon rank-sum test. The top 10 mutated genes in subgroups were analyzed using the “maftools” R package. The mutated rates of specific genes between subgroups were compared using Chi-square test. The correlations of PHLDA2 expression with tumor mutational burden (TMB) and microsatellite instability (MSI) were explored by Pearson relative analysis.

DNA methylation data from the TCGA database were analyzed. The methylation level of probes located in PHLDA2 between tumor and normal tissues, and tumors with different pTNM stages, was compared by Wilcoxon rank-sum test. Pearson relative analysis was performed to explore the correlation of PHLDA2 expression and methylation level of probes in ccRCC. KM survival analysis was conducted for OS to explore the association of methylation level of probes with prognosis.

### Functional enrichment analysis

The “DESeq2” R package was utilized to analyze the differential expressed genes (DEG) between subgroups. Genes with an absolute fold change > 1 and adjusted *p* < 0.05 were defined as statistically significant DEGs. Gene Ontology (GO) and Kyoto Encyclopedia of Genes and Genomes (KEGG) analysis using the “clusterProfiler” and “org.Hs.eg.db” R packages was performed. Gene set enrichment analysis (GSEA) was conducted using the GSEA software version 4.3.2. Genesets with an absolute normalized enrichment score > 1, a *p* < 0.05, and a false discovery rate < 0.05 were identified as profoundly enriched. The enrichment of hallmark pathways calculated by single sample GSEA (ssGSEA) in the CheckMate025 cohort was analyzed to verify the results derived from the TCGA database.

### Immune-related analysis

The Cibersort algorithm was applied to evaluate the correlation between PHLDA2 expression and 22 types of cells in the TCGA database and the West China Hospital cohort. Infiltration level of cells calculated utilizing xCell algorithm in both the CheckMate025 and the IMmotion151 cohorts was analyzed to verify the results derived from the TCGA database. Pearson relative analysis was performed to explore the correlations between PHLDA2 expression and markers of T cell exhaustion, cancer-associated fibroblasts (CAF). Wilcoxon rank-sum test was utilized to compare the expression level of Treg markers, immune checkpoints between groups. Responses for immunotherapy between subgroups were predicted using TIDE algorithm, a computational method to model T cell exhaustion and exclusion [27].

### Validations of therapeutic efficacy

The transcriptomic data of three RCTs were obtained. CheckMate025 (NCT01668784) is a phase 3 study comparing nivolumab with everolimus in advanced ccRCC patients who had received previous treatment [5]. IMmotion151 (NCT02420821) is a phase 3 study comparing first-line atezolizumab plus bevacizumab versus sunitinib in patients with previously untreated metastatic RCC [6]. Patients with non-ccRCC were excluded from the analysis. JAVELIN101 (NCT02684006) is a phase 3 study comparing avelumab plus axitinib with sunitinib in patients with advanced ccRCC [7]. Patients were stratified according to the median value of PHLDA2 expression. KM survival analysis was performed to compare the OS and progression-free survival (PFS) of patients.

## Results

### All PHLDA family members were aberrantly up-regulated in ccRCC

**Fig. 1:**
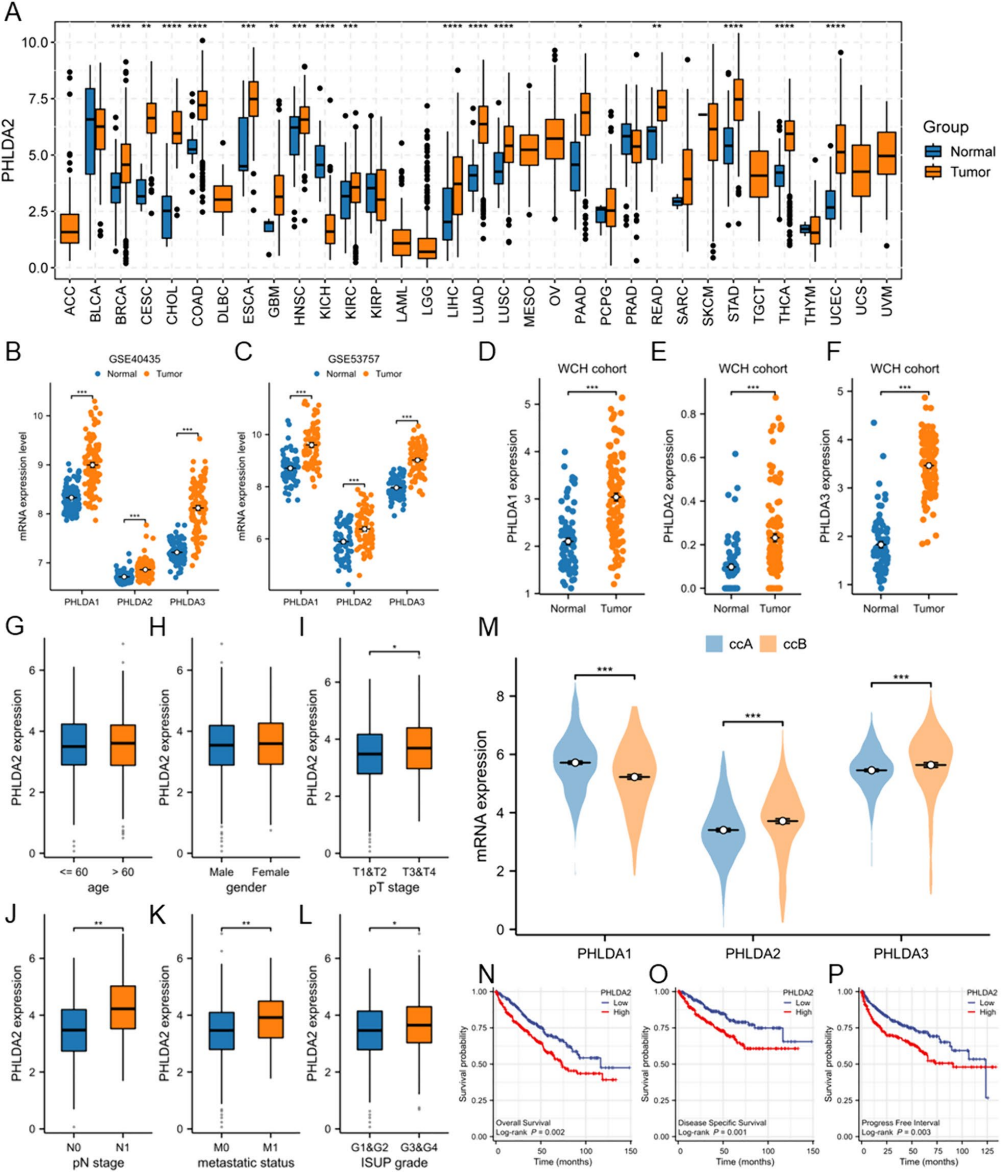
Expression pattern and correlations with clinicopathologic parameters and survival of PHLDA family members in clear cell renal cell carcinoma (ccRCC). **A** Expression level of PHLDA2 between tumor and normal tissues in pan-cancer in the TCGA database. *p < 0.05, **p < 0.01, ***p < 0.001. ****p < 0.0001. **B** Expression level of PHLDA1, PHLDA2, PHLDA3 between tumor (*n* = 101) and normal tissues (*n* = 101) in ccRCC in GSE40435. **C** Expression level of PHLDA1, PHLDA2, PHLDA3 between tumor (*n* = 72) and normal tissues (*n* = 72) in ccRCC in GSE53757. **D**–**F** Expression level of PHLDA1, PHLDA2, PHLDA3 between tumor (*n* = 117) and normal tissues (*n* = 79) in ccRCC in West China Hospital cohort. **G**–**L** Correlations between PHLDA2 expression level and clinicopathologic parameters of ccRCC, including age, gender, pT stage, pN stage, metastatic status, ISUP grade. (M) Expression level of PHLDA1, PHLDA2, PHLDA3 between ccA and ccB subtype of ccRCC. **N**–**P** Associations between PHLDA2 expression and OS, DSS, PFI of ccRCC patients by Kaplan–Meier survival analysis

Firstly, we assessed differences in expression patterns of PHLDA family members between tumor and normal tissues in pan-cancer to investigate the potential role of PHLDA family members in tumorigenesis. The results illustrated that PHLDA1 was up-regulated in COAD, GBM, KICH, KIRC, LUSC, READ, STAD (all *p* < 0.05), while down-regulated in BLCA, BRCA, KIRP, LIHC. PRAD, THCA (all *p* < 0.05) (Supplementary Fig. 1A). The significantly higher expression of PHLDA2 was observed in multiple cancers, including BRCA, CESC, CHOL, COAD, ESCA, GBM, HNSC, KIRC, LIHC, LUAD, LUSC, PAAD, READ, STAD, THCA, UCEC (all *p* < 0.05), and lower expression of PHLDA2 was only observed in KICH (*p* < 0.05) (Fig. 1A). The expression of PHLDA3 was elevated in BLCA, CHOL, COAD, ESCA, HNSC, KIRC, KIRP, LIHC, PCPG, THCA, THYM (all *p* < 0.05), and was decreased in BRCA, KICH, PRAD (all *p* < 0.05) (Supplementary Fig. 1B).

We then focused on ccRCC, and obtained microarray data from two GEO data sets to validate the expression profile of PHLDA family members. Unsurprisingly, a notable pattern of elevated PHLDA1, PHLDA2 and PHLDA3 expression in tumor tissues was observed in both GSE40435 and GSE53757 (all *p* < 0.05) (Fig. 1B, C). Furthermore, we performed RNA-seq on 117 treatment-naïve ccRCC primary tumors and 79 adjacent renal tissues collected from West China Hospital, Sichuan University and compared the expression level of PHLDA family members between tumor and normal tissues, yielding consistent results (all *p* < 0.05) (Fig. 1D–F).

### The expression of PHLDA2 but not PHLDA1 or PHLDA3 could be served as an independent prognostic factor in ccRCC

To explore the clinical significance of PHLDA family expression in ccRCC, we collected clinicopathologic parameters, including age, gender, pT stage, pN stage, metastatic status, and ISUP grade, and analyzed the association of PHLDA family expression with these features. Regarding PHLDA1, negative correlations were observed between PHLDA1 expression level and metastatic status, ISUP grade (both *p* < 0.05) (Supplementary Fig. 1C–H). On the opposite, significant positive correlations were found between PHLDA2 expression level and pT stage, pN stage, metastatic status, ISUP grade (all *p* < 0.05) (Fig. 1G–L). Similarly, for PHLDA3, positive correlations were observed between PHLDA3 expression level and pN stage, metastatic status and ISUP grade (all *p* < 0.05) (Supplementary Fig. 1I–N). Besides, the expression level of PHLDA3 differed between genders, with an elevation observed in male patients with ccRCC (*p* < 0.05). In addition, we classified ccRCC samples from the TCGA database into good risk ccA and poor risk ccB subtypes using a well-established 34-gene signature. To our interest, PHLDA1 expression was higher in ccA subtype, while conversely, we found higher expression of both PHLDA2 and PHLDA3 in ccB subtype (Fig. 1M).

Further analysis was conduct to comprehend how PHLDA family influences patient prognosis in ccRCC. KM survival analysis in the TCGA ccRCC cohort revealed that higher PHLDA1 expression was associated with better OS (*p* < 0.05), DSS (*p* < 0.05) and PFI (*p* = 0.054) (Supplementary Fig. 1O–Q). On the contrary, higher expression of PHLDA2 was associated with poorer OS, DSS and PFI (all *p* < 0.05) (Fig. 1N–P). For PHLDA3, no correlations were observed between expression level and OS, DSS (Supplementary Fig. 1R, S). However, higher PHLDA3 expression could still predict a worse PFI (*p* < 0.05) (Supplementary Fig. 1T).

To investigate whether expression of PHLDA family could serve as an independent prognostic factor in ccRCC, univariate and multivariate Cox regression analysis was then performed. For OS, besides age, pTNM stage and ISUP grade, only PHLDA2 expression, but not PHLDA1 or PHLDA3 expression, was validated as an independent prognostic factor, with lower expression level predicting a favorable outcome (HR 1.293, 95% CI 1.067–1.567, *p* < 0.01) (Table 1). In addition, the role of only PHLDA2 expression as an independent predictor for DSS (HR 1.585, 95% CI 1.238–2.029, *p* < 0.001) (Table 2) and PFI (HR 1.304, 95% CI 1.059–1.604, *p* < 0.05) (Table 3) in ccRCC was confirmed.

**Table 1:** Univariate and multivariate Cox regression analysis of OS in ccRCC patients

Characteristics	Total (*N*)	Univariate analysis			Multivariate analysis	
		Hazard ratio (95% CI)	*p* value		Hazard ratio (95% CI)	*p* value
Gender	541					
Male	354	Reference				
Female	187	1.083 (0.796–1.473)	0.613			
Age	541					
< = 60	269	Reference			Reference	
> 60	272	1.791 (1.319–2.432)	**< 0.001**		1.534 (1.126–2.088)	**0.007**
Pathologic stage	538					
Stage I & Stage II	332	Reference			Reference	
Stage III & Stage IV	206	3.910 (2.852–5.360)	**< 0.001**		3.060 (2.185–4.286)	**< 0.001**
Histologic grade	533					
G1&G2	250	Reference			Reference	
G3 & G4	283	2.665 (1.898–3.743)	**< 0.001**		1.701 (1.184–2.444)	**0.004**
PHLDA1	541	0.850 (0.735–0.982)	**0.028**		0.879 (0.750–1.030)	0.111
PHLDA2	541	1.245 (1.077–1.439)	**0.003**		1.253 (1.054–1.488)	**0.010**
PHLDA3	541	1.182 (0.999–1.399)	0.052		0.897 (0.736–1.093)	0.282

**Table 2:** Univariate and multivariate Cox regression analysis of DSS in ccRCC patients

Characteristics	Total (*N*)	Univariate analysis			Multivariate analysis	
		Hazard ratio (95% CI)	*p* value		Hazard ratio (95% CI)	*p* value
Gender	530					
Male	349	Reference				
Female	181	0.845 (0.562–1.272)	0.420			
Age	530					
< = 60	265	Reference				
> 60	265	1.351 (0.926–1.971)	0.118			
Pathologic stage	527					
Stage I & Stage II	329	Reference			Reference	
Stage III & Stage IV	198	9.937 (5.989–16.486)	**< 0.001**		7.282 (4.288–12.367)	**< 0.001**
Histologic grade	522					
G1 & G2	249	Reference			Reference	
G3 & G4	273	4.850 (2.925–8.043)	**< 0.001**		2.371 (1.396–4.029)	**0.001**
PHLDA1	530	0.774 (0.645–0.928)	**0.006**		0.817 (0.663–1.006)	0.057
PHLDA2	530	1.438 (1.194–1.731)	**< 0.001**		1.485 (1.190–1.853)	**< 0.001**
PHLDA3	530	1.317 (1.054–1.644)	**0.015**		0.867 (0.669–1.123)	0.279

**Table 3:** Univariate and multivariate Cox regression analysis of PFI in ccRCC patients

Characteristics	Total (*N*)	Univariate analysis			Multivariate analysis	
		Hazard ratio (95% CI)	*p* value		Hazard ratio (95% CI)	*p* value
Gender	539					
Male	353	Reference			Reference	
Female	186	0.677 (0.479–0.959)	**0.028**		0.650 (0.456–0.926)	**0.017**
Age	539					
< = 60	268	Reference				
> 60	271	1.285 (0.942–1.754)	0.114			
Pathologic stage	536					
Stage I & Stage II	332	Reference			Reference	
Stage III & Stage IV	204	6.877 (4.813–9.826)	**< 0.001**		5.454 (3.746–7.939)	**< 0.001**
Histologic grade	531					
G1 & G2	250	Reference			Reference	
G3 & G4	281	3.684 (2.530–5.364)	**< 0.001**		2.046 (1.380–3.032)	**< 0.001**
PHLDA1	539	0.860 (0.743–0.996)	**0.044**		0.905 (0.766–1.070)	0.242
PHLDA2	539	1.340 (1.152–1.559)	**< 0.001**		1.277 (1.063–1.536)	**0.009**
PHLDA3	539	1.425 (1.181–1.720)	**< 0.001**		1.031 (0.833–1.275)	0.781

### PHLDA2 overexpression was associated with DNA hypomethylation

**Fig. 2:**
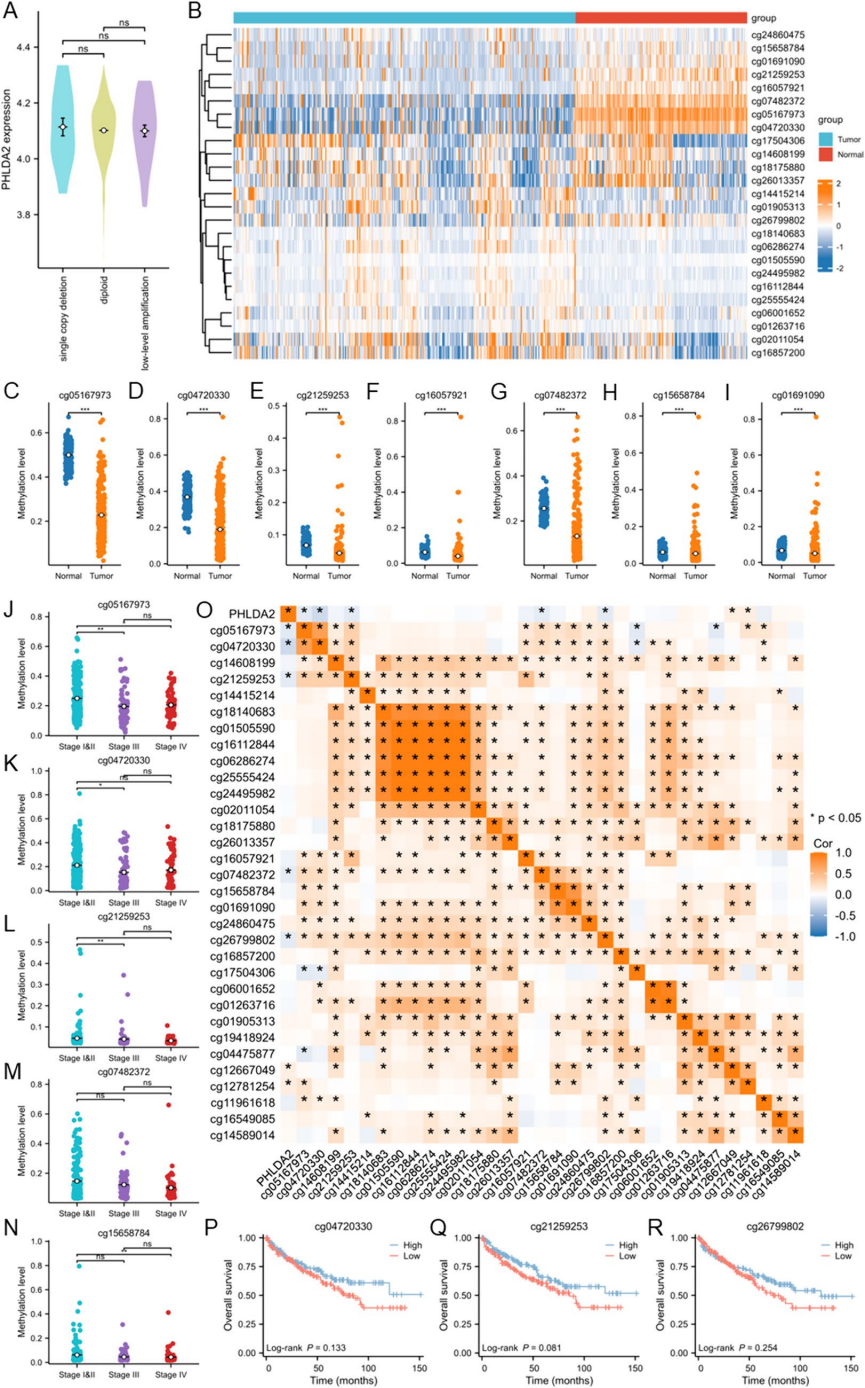
Up-regulation of PHLDA2 was associated with aberrant DNA hypomethylation in ccRCC. **A** Expression level of PHLDA2 among tumors with different status of copy number variation, including single copy deletion, diploid, low-level amplification. **B** Heatmap showed DNA methylation level of all probes in PHLDA2 between tumor and normal tissues in ccRCC. **C**–**I** DNA methylation level of cg05167973, cg04720330, cg21259253, cg16057921, cg07482372, cg15658784, cg01691090 between tumor and normal tissues in ccRCC. ***p < 0.001. **J**–**N** DNA methylation level of cg05167973, cg04720330, cg21259253, cg07482372, cg15658784 among ccRCC with different pTNM stages. *p < 0.05, **p < 0.01. **O** Heatmap illustrated correlations of PHLDA2 expression with DNA methylation level of all probes in PHLDA2 in ccRCC. **P**–**R** Associations between DNA methylation level of cg04720330, cg21259253, cg26799802 and OS of ccRCC patients by Kaplan–Meier survival analysis

Since only PHLDA2 could be served as an independent prognostic factor in ccRCC, we then focused on PHLDA2 to explore its role in ccRCC combining multi-omics data. We initially detected the mutation and copy number variation status to find out the mechanism underlying the up-regulation of PHLDA2 in ccRCC. However, no mutation of PHLDA2 was found in TCGA ccRCC cohort. Besides, no correlation was found between copy number and expression level of PHLDA2 (Fig. 2A). Taken together, the results suggested that genetic alteration could not explain the dysregulation of PHLDA2 in ccRCC.

Next, the methylation status of PHLDA2 was examined. Compared to normal tissues, multiple probes demonstrated decreased methylation level in cccRCC, including cg05167973, cg04720330, cg21259253, cg16057921, cg07482372, cg15658784, cg01691090 (*p* < 0.05) (Fig. 2B–I). Besides, the methylation levels of cg05167973, cg04720330, cg21259253, cg07482372, cg15658784 (*p* < 0.05) were positively correlated with pTNM stage in ccRCC (Fig. 2J–N). In addition, we investigated the correlations between methylation levels of probes and PHLDA2 expression. It turned out that lower level of methylation level in cg05167973 (*r* = − 0.14, *p* < 0.05), cg04720330 (*r* = − 0.24, *p* < 0.05), cg21259253 (*r* = − 0.13, *p* < 0.05), cg07482372 (*r* = − 0.13, *p* < 0.05) and cg26799802 (*r* = − 0.13, *p* < 0.05) was associated with increased PHLDA2 expression, indicating that elevated expression of PHLDA2 in ccRCC could be attributed to methylation modification (Fig. 2O). Moreover, hypomethylation of cg04720330 (*p* = 0.133), cg21259253 (*p* = 0.081) and cg26799802 (*p* = 0.254) indicated poorer OS in ccRCC, although without statistical significance (Fig. 2P–R).

**Fig. 3:**
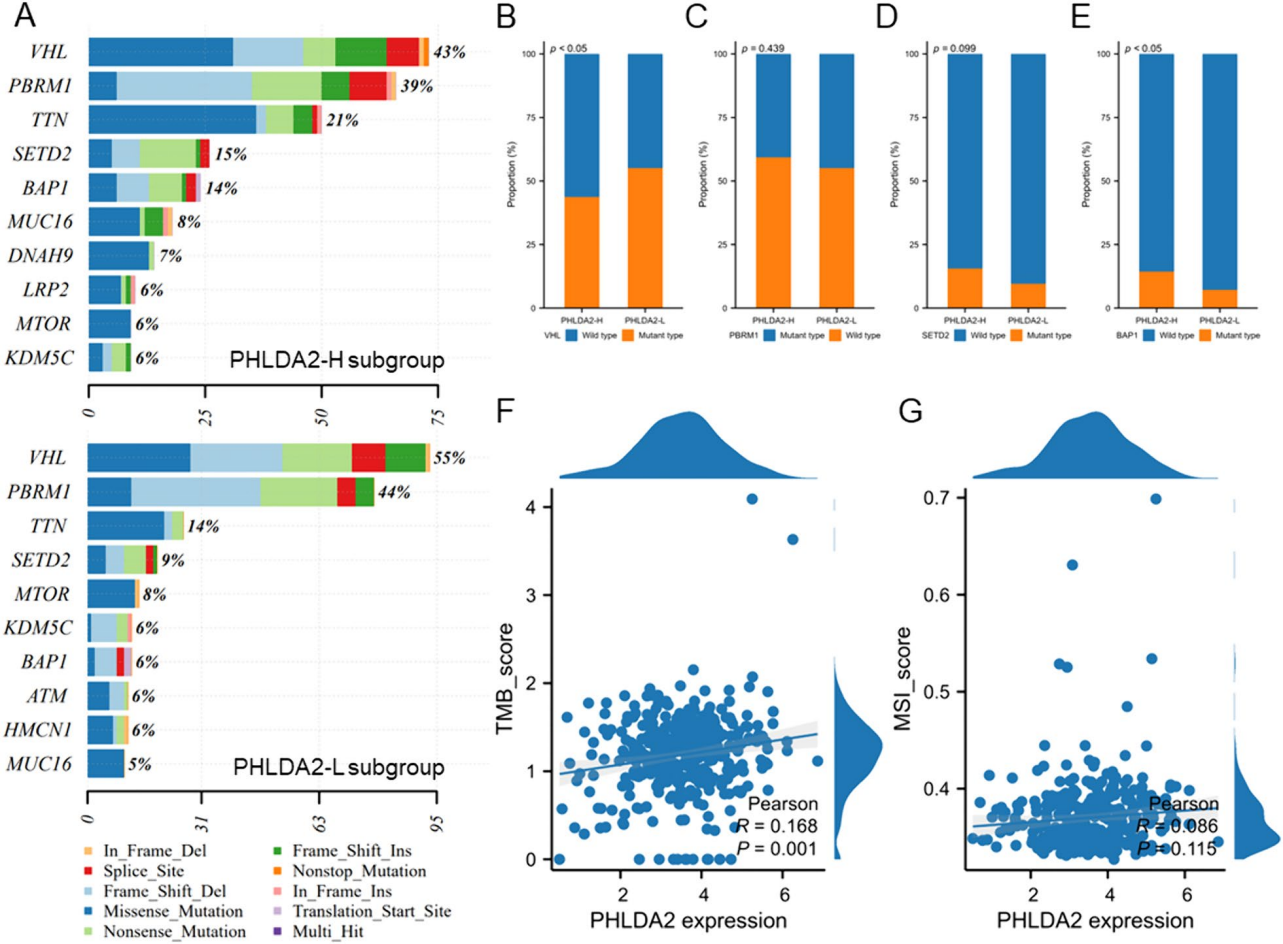
Distinct mutational landscapes between PHLDA2-H and PHLDA2-L subgroups. **A** Top 10 mutated genes in PHLDA2-H and PHLDA2-L subgroups in ccRCC. **B**–**E** Comparisons of VHL, PBRM1, SETD2, BAP1 mutation rate between PHLDA2-H and PHLDA2-L subgroups in ccRCC. **F** Correlation between PHLDA2 expression and tumor mutational burden in ccRCC. **G** Correlation between PHLDA2 expression and microsatellite instability in ccRCC

Eventually, we divided ccRCC samples into PHLDA2-high (PHLDA2-H) and PHLDA2-low (PHLDA2-L) subgroups based on stratification by median expression of PHLDA2, and compared the mutational landscape between the two subgroups. The results illustrated that in the PHLDA2-H subgroup, the most frequently mutated genes were VHL (43%), followed by PBRM1 (39%), TTN (21%), SETD2 (15%), BAP1 (14%) (Fig. 3A). In the PHLDA2-L subgroup, the top five most frequently mutated genes were VHL (55%), PBRM1 (44%), TTN (14%), SETD2 (9%) and MTOR (8%) (Fig. 3A). We further compared the mutation rate of 4 genes (VHL, PBRM1, SETD2, BAP1) which play essential roles in the development of ccRCC between PHLDA2-H and PHLDA2-L subgroups (Fig. 3B–E). Interestingly, VHL mutations were more enriched in PHLDA2-L subgroup compared to PHLDA2-H subgroup (55% vs. 43%, *p* < 0.05). On the contrary, PHLDA2-L subgroup had lower SETD2 (9% vs. 14%, *p* = 0.099) and BAP1 (8% vs. 14%, *p* < 0.05) mutation rate compared to PHLDA2-H subgroup. Consistently, higher expression of PHLDA2 was observed in BAP1-mutated patients (*p* < 0.05) and SETD2-mutated patients (*p* = 0.06), compared to patients with wild type BAP1 or SETD2, respectively (Supplementary Fig. 2A–D). In addition, PHLDA2 expression was positively correlated with TMB score (*r* = 0.168, *p* = 0.001) but was not associated with MSI score (*r* = 0.086. *p* > 0.05) (Fig. 3F, G).

### ECM, cell cycle, and immune-related pathways were enriched in PHLDA2-H subgroup

**Fig. 4:**
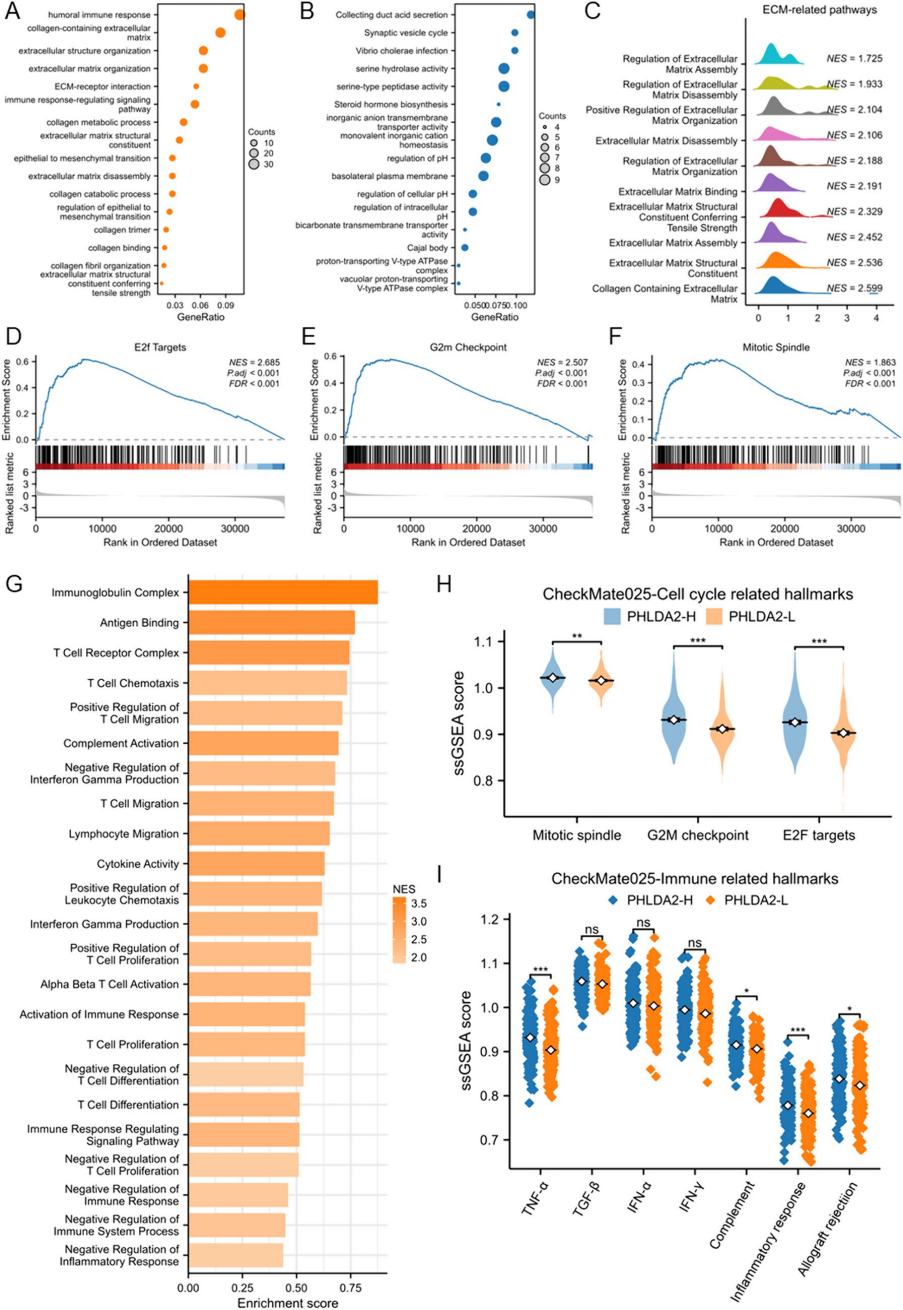
ECM, cell cycle and immune-related pathways were enriched in PHLDA2-H subgroup. **A** KEGG pathway and GO term enrichment analysis of genes up-regulated in PHLDA2-H subgroup compared with PHLDA2-L subgroup in ccRCC. **B** KEGG pathway and GO term enrichment analysis of genes down-regulated in PHLDA2-H subgroup compared with PHLDA2-L subgroup in ccRCC. **D**–**F** Enrichment of cell cycle-related pathways, including E2F targets, G2M checkpoint, mitotic spindle in PHLDA2-H subgroup in ccRCC by GSEA. **G** Enrichment of immune-related pathways in PHLDA2-H subgroup in ccRCC by GSEA. **H** Enrichment of cell cycle-related pathways, including E2F targets, G2M checkpoint, mitotic spindle in PHLDA2-H subgroup in ccRCC by ssGSEA in the CheckMate025 cohort. **I** Enrichment of immune-related pathways, including TNF-α, complement, inflammatory response, allograft rejection in PHLDA2-H subgroup in ccRCC by ssGSEA in the CheckMate025 cohort. *p < 0.05, **p < 0.01, ***p < 0.001

The difference of transcriptomic characteristics between PHLDA2-H and PHLDA2-L subgroups was further explored. In TCGA ccRCC cohort, compared with PHLDA2-L subgroup, we identified a total of 3212 differential expressed genes (DEGs) in PHLDA2-H subgroup, of which 712 were up-regulated, and 2500 were down-regulated (Supplementary Table 1). KEGG pathway and GO term enrichment analyses were performed to illustrate the function roles of these genes. The results revealed that DEGs up-regulated in PHLDA2-H subgroup were significantly enriched in ECM-related pathways, including ECM organization, extracellular structure organization, collagen metabolic process, collagen catabolic process (Fig. 4A). Besides, EMT-related pathways were enriched. DEGs down-regulated in PHLDA2-H subgroup were remarkably enriched in regulation of PH, monovalent inorganic cation homeostasis, etc. (Fig. 4B). Next, GSEA was conducted, and the results revealed that multiple pathways related to ECM remodeling were up-regulated in PHLDA2-H subgroup, which was consistent with the results above (Fig. 4C). Cell cycle-related pathways, including E2F targets, G2M checkpoint, mitotic spindle, were also enriched in PHLDA2-H subgroup (Fig. 4D–F). Moreover, various immune-related pathways, in which both positive and negative regulations of the immune system were observed, were up-regulated as well, reflecting the complicated nature of the TME in PHLDA2-H subgroup of ccRCC (Fig. 4G).

Next, we utilized hallmark gene set scores calculated by performing ssGSEA on transcriptomic data from CheckMate025, a randomized trial of nivolumab versus everolimus in patients with metastatic ccRCC. Similarly, the results demonstrated that the ssGSEA scores of E2F targets, G2M checkpoint, mitotic spindle, along with immune-related hallmarks, were enriched in PHLDA2-H subgroup (Fig. 4H, I).

### Immunosuppressive microenvironment in PHLDA2-H subgroup might impair the efficacy of immunotherapy

**Fig. 5:**
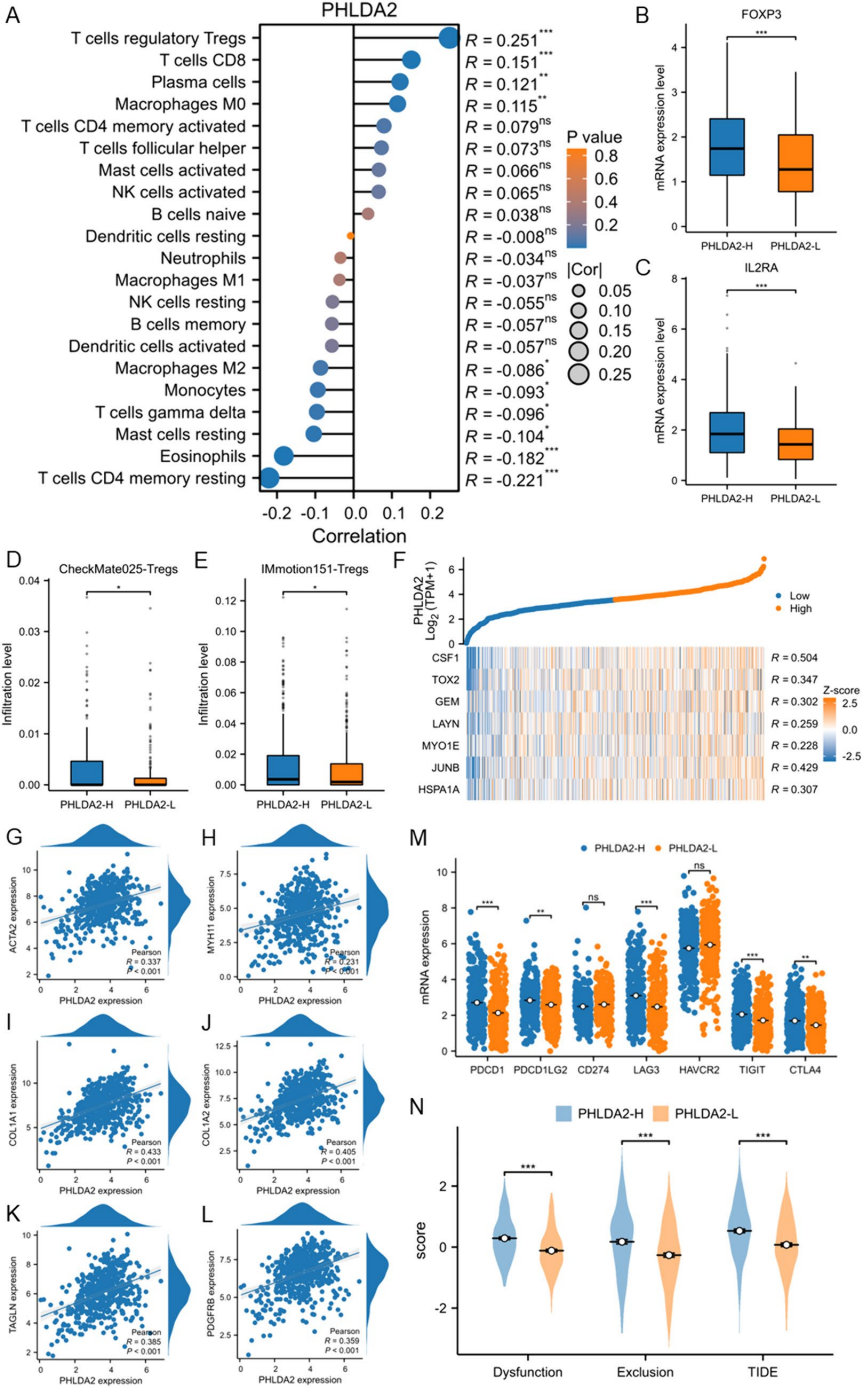
Immunosuppressive microenvironment in PHLDA2-H subgroup might impair the efficacy of immunotherapy. **A** Correlations between PHLDA2 expression and infiltration level of different immune cells in ccRCC by Cibersort algorithm. *p < 0.05, **p < 0.01, ***p < 0.001. **B**, **C** Expression level of Treg markers, including FOXP3 and IL2RA, between PHLDA2-H and PHLDA2-L subgroups in ccRCC. **D** Infiltration level of Tregs between PHLDA2-H and PHLDA2-L subgroups in ccRCC by xCell algorithm in the CheckMate025 cohort. **E** Infiltration level of Tregs between PHLDA2-H and PHLDA2-L subgroups in ccRCC by xCell algorithm in the IMmotion151 cohort. **F** Correlations between PHLDA2 expression and expression of terminal exhaustion markers, including CSF1, TOX2, GEM, LAYN, MYO1E, and early exhaustion markers, including JUNB, HSPA1A, in ccRCC. **G**–**L** Correlations between PHLDA2 expression and expression of CAF markers in ccRCC. **M** Comparisons of expression of immune checkpoints between PHLDA2-H and PHLDA2-L subgroups in ccRCC. **N** Comparisons of T cell dysfunction score, T cell exclusion score and TIDE score between PHLDA2-H and PHLDA2-L subgroups in ccRCC

Afterwards, we focused on the characteristics of TME between the two subgroups in the TCGA ccRCC cohort. The infiltration level of various immune cells was first estimated based on CIBERSORT algorithm (Fig. 5A). The results reflected a negative correlation of PHLDA2 expression with infiltration of T cells CD4 memory resting (*r* = − 0.221, *p* < 0.001), eosinophils (*r* = − 0.182, *p* < 0.001), mast cells resting (*r* = − 0.104, *p* < 0.05). Among the immune cells the infiltration levels of which were positively correlated with PHLDA2 expression, Tregs showed the strongest positive correlation (*r* = 0.251, *p* < 0.001). Regarding that Tregs play a crucial role in immunosuppression, we then gave emphasis to this subpopulation. Exactly, elevated expression of classical Treg markers, including FOXP3 and IL2RA, were detected in PHLDA2-H subgroup (*p* < 0.001) (Fig. 5B, C). Moreover, the enhanced recruitment of Tregs in PHLDA2-H subgroup was confirmed using data from CheckMate025 and IMmotion151 (Fig. 5D, E). Actually, Tregs trigger T cell exhaustion, thereby mediate the suppression of anti-tumor immunity. Based on this point, we then evaluated the degree of T cell exhaustion between the two subgroups. Strong positive correlations were observed between the expression level of PHLDA2 and multiple terminal exhaustion markers, including CSF1 (*r* = 0.504, *p* < 0.001), TOX2 (*r* = 0.347, *p* < 0.001), GEM (*r* = 0.302, *p* < 0.001)), LAYN (*r* = 0.259, *p* < 0.001), MYO1E (*r* = 0.228, *p* < 0.001), and early exhaustion markers, including JUNB (*r* = 0.429, *p* < 0.001), HSPA1A (*r* = 0.307, *p* < 0.001) (Fig. 5F). Given the results that ECM-related pathways were up-regulated in PHLDA2-H subgroup, in which cancer associated fibroblast (CAF) plays a vital role, we then investigated the association between PHLDA2 and CAF, and observed positive correlations between the expression level of PHLDA2 and all CAF markers, including ACTA2 (*r* = 0.337, *p* < 0.001), MYH11 (*r* = 0.231, *p* < 0.001), COL1A1 (*r* = 0.433, *p* < 0.001), COL1A2 (*r* = 0.405, *p* < 0.001), TAGLN (*r* = 0.385, *p* < 0.001), PDGFRB (*r* = 0.359, *p* < 0.001) (Fig. 5G–L). In addition, the expression level of several immune checkpoints was explored, and we found higher expression level of PDCD1, PDCD1LG2, LAG3, TIGIT, CTLA4 in PHLDA2-H subgroup (*p* < 0.05) (Fig. 5M).

Ultimately, we calculated and compared the TIDE scores, which were used to evaluate the immunotherapy predictive efficacy, of PHLDA2-H and PHLDA2-L subgroup. In contrast to the PHLDA2-L subgroup, both the dysfunction score and the exclusion score, and the integrated TIDE score of PHLDA2-H subgroup were much higher (all *p* < 0.001), indicating the poor response of PHLDA2-H subgroup to ICI-based treatment (Fig. 5N).

**Fig. 6:**
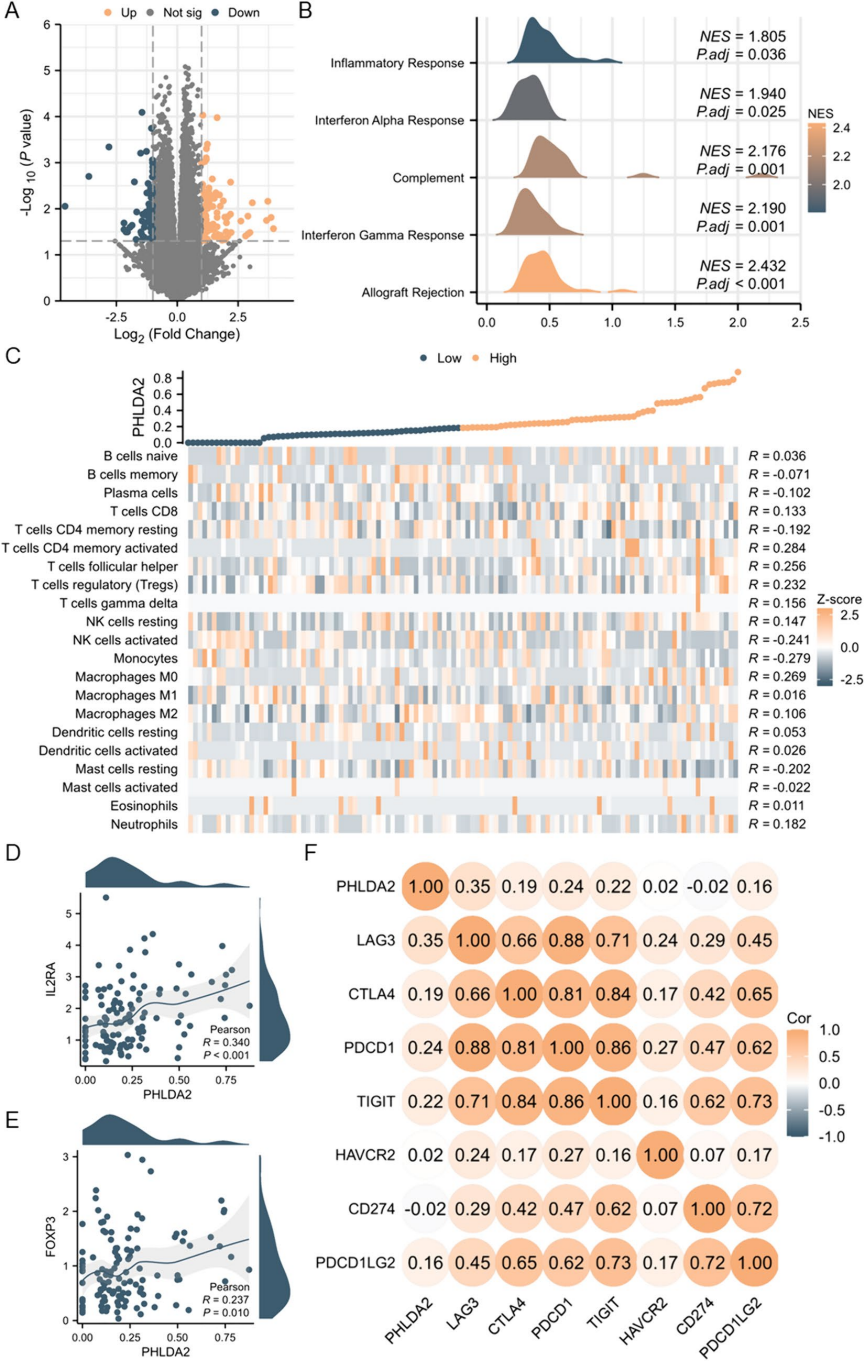
Validation of an immunosuppressive microenvironment in PHLDA2-H subgroup in ccRCC in West China Hospital cohort. **A** Volcano plot showed differential expressed genes between PHLDA2-H and PHLDA2-L subgroups in ccRCC in West China Hospital cohort. **B** Enrichment of immune-related pathways, including allograft rejection, IFN-γ, complement, IFN-α, inflammatory response, in PHLDA2-H subgroup in ccRCC in West China Hospital cohort. **C** Correlations between PHLDA2 expression and infiltration level of different immune cells in ccRCC by Cibersort algorithm in West China Hospital cohort. **D**, **E** Correlations between PHLDA2 expression and Treg markers, including IL2RA and FOXP3, in ccRCC in West China Hospital cohort. **F** Correlations between PHLDA2 expression and expression of immune checkpoints in ccRCC in West China Hospital cohort

We further explored our transcriptomic data of 117 primary ccRCC tumors to validate the immunosuppressive features of TME in PHLDA2-H subgroup. Samples were divided into PHLDA2-H and PHLDA2-L subgroups by the median value of PHLDA2 expression and the DEGs between the two subgroups were identified (Fig. 6A). GSEA showed enrichment of immune-related pathways, including allograft rejection, IFN-γ response, complement, IFN-α, inflammatory response, in PHLDA2-H subgroup (Fig. 6B). Cibersort algorithm was applied to calculate the infiltration level of immune cells, and positive correlation was observed between PHLDA2 expression and infiltration level of Tregs (*r* = 0.232) and markers of Tregs, IL2RA (*r* = 0.34), FOXP3 (*r* = 0.237) (all *p* < 0.05) (Fig. 6C–E). The association of PHLDA2 expression with immune checkpoints was further investigated, and positive correlation was observed, especially LAG3 (*r* = 0.35, *p* < 0.05) (Fig. 6F). In general, results derived from our transcriptomic data was highly consistent with those derived from the TCGA database, further confirming an immunosuppressive TME in the PHLDA2-H subgroup of ccRCC.

### Elevated PHLDA2 expression could predict the therapeutic effects of ICI plus TKI combination therapy in ccRCC

To verify whether the differences in immune phenotypes of TME between PHLDA2-H and PHLDA2-L subgroups could accurately predict the efficacy of immunotherapy, we employed data from three RCT, namely CheckMate025, IMmotion151 and JAVELIN101, to explore the predictive role of PHLDA2.

**Fig. 7:**
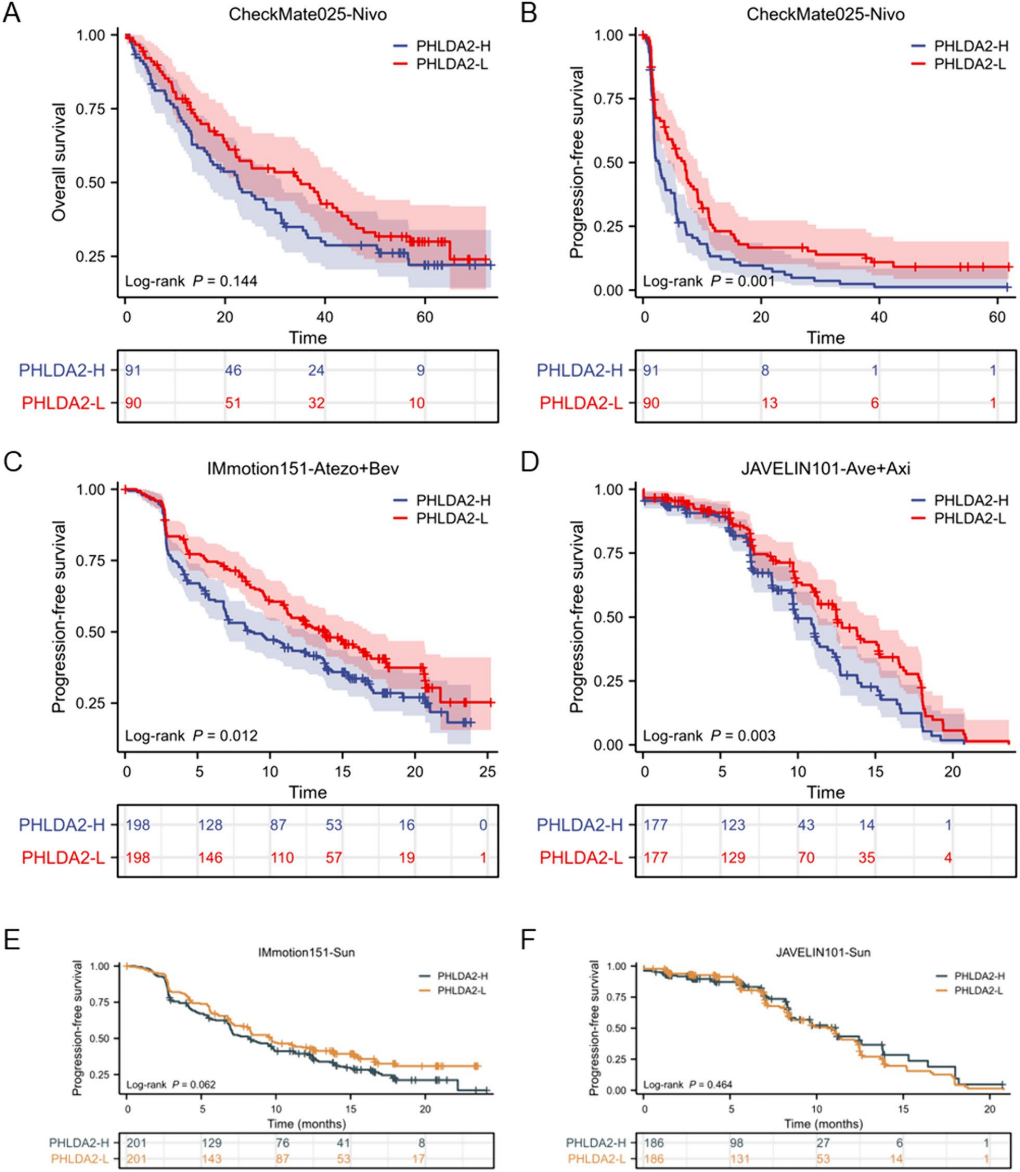
Elevated PHLDA2 expression could predict the therapeutic effects of ICI plus TKI combination therapy in ccRCC. **A** Associations between PHLDA2 expression and OS of metastatic ccRCC patients in the nivolumab arm in the CheckMate025 cohort. **B** Associations between PHLDA2 expression and PFS of metastatic ccRCC patients in the nivolumab arm in the CheckMate025 cohort. **C** Associations between PHLDA2 expression and PFS of metastatic ccRCC patients in the atezolizumab plus bevacizumab arm in the IMmotion151 cohort. **D** Associations between PHLDA2 expression and PFS of metastatic ccRCC patients in the avelumab plus axitinib arm in the JAVELIN101 cohort. **E** Associations between PHLDA2 expression and PFS of metastatic ccRCC patients in the sunitinib arm in the IMmotion151 cohort. **F** Associations between PHLDA2 expression and PFS of metastatic ccRCC patients in the sunitinib arm in the JAVELIN101 cohor

In CheckMate025 cohort, patients with advanced ccRCC for which they had received previous treatment with one or two regimens of anti-angiogenic treatment were enrolled, and those receiving nivolumab were selected for further analyzed. We grouped the patients into PHLDA2-H and PHLDA2-L subgroup according to the median expression of PHLDA2. Inevitably, both the OS (*p* = 0.144) and the PFS (*p* = 0.001) of PHLDA2-H subgroup treated with nivolumab were worse (Fig. 7A, B). Furthermore, patients in PHLDA2-L subgroup had better objective response rate (ORR) (*p* = 0.175) and disease control rate (DCR) (*p* < 0.05) than those in PHLDA2-H subgroup (Supplementary Fig. 3A, B). The expression level of PHLDA2 was higher in patients who did not reach a DCR (*p* < 0.05) (Supplementary Fig. 3C).

Although combinations of immunotherapy with anti-angiogenic agents as first-line therapy significantly improve outcomes of metastatic RCC patients, there still exists patients who cannot benefit from this regimen. We then further explored whether PHLDA2 expression could predict the therapeutic efficacy of combination therapy. In IMmotion151 cohort, we selected patients with metastatic ccRCC receiving first-line atezolizumab plus bevacizumab and grouped them into PHLDA2-H and PHLDA2-L subgroups. As expected, PHLDA2-H subgroup achieved poorer PFS than PHLDA2-L subgroup (*p* = 0.012) (Fig. 7C). In PHLDA2-L subgroup, higher proportion of patients with disease control was observed (*p* = 0.103) (Supplementary Fig. 3D). In JAVELIN101 cohort, patients with advanced ccRCC receiving avelumab plus axitinib as first-line treatment were grouped into PHLDA2-H and PHLDA2-L subgroup, and we observed poorer PFS in PHLDA2-H subgroup, which was consistent with the results from IMmotion151 cohort (*p* = 0.003) (Fig. 7D). However, no difference in PFS was found between PHLDA2-H and PHLDA2-L subgroups among patients receiving first-line sunitinib in both IMmotion151 and JAVELIN101 cohorts (Fig. 7E, F). The role of PHLDA1 and PHLDA3 expression in predicting therapeutic efficacy was also explored across the three cohort, without correlation findings (Supplementary Fig. 3E–P).

Taken together, PHLDA2 expression can be served specially as a robust predictive biomarker for immunotherapy plus anti-angiogenic agent combination in ccRCC.

## Discussion

Over the past decade, the therapeutic landscape of metastatic ccRCC has undergone rapid evolution. The application of ICIs plus anti-angiogenic agents represents a new standard of care for the first-line treatment of ccRCC, and has prolonged the survival of patients with metastatic ccRCC. Nevertheless, due to the heterogeneity of ccRCC, about 6–20% of patients experience primary resistance, and durable effects are only observed in a limited subset of patients. Hence, identifying robust biomarkers to predict the optimal candidates for ICI plus anti-angiogenic agent combination therapy in ccRCC is urgently needed.

PHLDA2, one crucial member of the PHLDA family encoding proteins containing PH domains which are highly conserved throughout eukaryotes, is located in a cluster of imprinted genes on chromosome 11p15.5, with preferential expression from the maternal allele in placenta. Previous studies demonstrated that elevated expression of PHLDA2 has been reported to be associated with fetal growth restriction [28]. Besides, PHLDA2 induced apoptosis, inhibited proliferation and led to inadequate invasion of trophoblast cells, suggesting an important role of PHLDA2 in the occurrence and progression of pregnancy-associated complications [29, 30]. In addition, over-expression of PHLDA2 has been reported to promote tumor development in multiple cancers. Knockdown of PHLDA2 activated apoptosis and autophagy, eventually causing inhibition of tumor growth through AKT/mTOR signaling in both colorectal cancer and glioma [18, 19]. Wang et al. revealed that in liver cancer, HSPA8 bound to the promoter of PHLDA2 to up-regulate its transcription through the coactivating transcription factor ETV4, and promoted the growth of tumor cells [17]. However, in certain cancers, PHLDA2 could also act as a tumor suppressor. In osteosarcoma, one type of aggressive bone tumor, PHLDA2 displayed decreased expression level, suggested a favorable prognosis for patients, and overexpressed PHLDA2 could impair tumorigenesis and metastasis both in vitro and in vivo [31, 32]. Thus, whether PHLDA2 functions as an oncogene or a tumor suppressor depends on the type of cancer. In this study, we observed for the first time that the expression level of PHLDA2 was up-regulated, consistent with the majority of cancers, including liver cancer, colorectal cancer, glioma, lung cancer [17–19, 33]. Furthermore, elevated PHLDA2 expression was associated with adverse clinicopathologic features and poorer survival in ccRCC. Thus, PHLDA2 could be served as a promising biomarker to predict the prognosis of ccRCC patients.

Epigenetic modifications, especially alterations in DNA methylation have been found in all types of cancers, including ccRCC [34, 35]. Hypomethylation of various tumor promoter genes has been identified in ccRCC. IL8, a chemokine that stimulates tumor cell proliferation and increases angiogenesis, was maximally hypomethylated in tumor tissue compared to normal tissue [36]. Cho et al. examined the methylation status of G250, which aids cancer progression by neutralizing the surrounding acidic pH, in ccRCC cell lines and normal kidney tissue samples, and demonstrated hypomethylation in the 5’ region [37]. In this study, the methylation status of PHLDA2 was investigated, and we found that, compared to normal tissues, several probes, including cg05167973, cg04720330, cg21259253, cg16057921, cg07482372, cg15658784, cg01691090, was hypomethylated in ccRCC. Besides, the expression of PHLDA2 was negatively correlated with methylation level of these probes, suggesting the potential role of DNA hypomethylation in aberrant up-regulation of PHLDA2 in ccRCC. Furthermore, hypomethylation of probes, for instance, cg04720330 and cg21259253, indicated higher pTNM stage and poorer OS in ccRCC. To our knowledge, no studies have investigated the role of DNA methylation alterations in PHLDA2 dysregulation in cancers, except for one study conducted by Fu and colleagues, which revealed that DNA methylation status of PHLDA2 in peripheral blood was associated with breast cancer susceptibility [38]. Our results reinforced the significance of DNA methylation alterations in ccRCC pathogenesis, pointing to the potential use of epigenetic modulators in the treatment of this malignancy.

The tumor microenvironment represents an intricate ecosystem which comprises tumor cells and a multitude of non-cancerous cells, including immune cells and stromal cells, and heavily affects disease biology and responses to systemic therapy [39–41]. ccRCC has been identified as an immunogenic tumor, characterized by rich infiltrates of T cells [42, 43]. Nonetheless, the majority of tumor-infiltrating T cells exhibited an immunosuppressive phenotype, characterized by high expression of multiple immune checkpoints, thus cannot mount anti-tumor responses effectively [42]. In addition, Gigante et al. revealed that CD8+ T cells from ccRCC patients expressed reduced levels of anti-apoptotic and proliferation-associated gene products when compared with normal donor T cells due to miR-29b and miR-198 overexpression, thus leading to immune dysfunction [44]. Multiple cell populations contribute to the immune evasion of tumor cells and suppression of T cell activation, among which Tregs play fundamental roles. Increased infiltration level of Tregs has been reported to predict worse prognosis across many cancers, and restrain therapeutic efficacy of ICI [45–49]. In this study, we also illustrated that PHLDA2 expression was positively correlated with Treg infiltrates in ccRCC in TCGA cohort, CheckMate025 cohort and IMmotion151 cohort. In addition, we observed pathways involved in ECM remodeling mainly enriched in PHLDA2-H subgroup. ECM components can establish an immunosuppressive microenvironment to stimulate tumor growth, and finally results in poor clinical response to immunotherapy [50]. To our interest, CAFs play vital role in ECM remodeling. Therefore, the association of PHLDA2 expression with CAFs was then explored and strong positive correlations were observed between the expression level of PHLDA2 and CAF markers, indicating higher infiltrates of CAFs in PHLDA2-H subgroup. It’s worth noting that ccRCC is characterized by metabolic dysregulation, for instance, hypoxia-inducible factor pathway and kynurenine pathway, which not only promote angiogenesis but also facilitate tumor growth and immune evasion [51, 52]. Previous studies have reported that PHLDA family members play vital roles in cancer metabolism, suggesting that in ccRCC, PHLDA2 may also shape an immunosuppressive microenvironment by regulating metabolism [53]. In sum, the results showed that elevated PHLDA2 expression was associated with a more immunosuppressive microenvironment in ccRCC, which might impair the efficacy of immunotherapy. Afterwards, our hypothesis was validated by three RCTs in ccRCC, namely CheckMate025, IMmotion151 and JAVELIN101. Regarding PHLDA2-H subgroup, we observed over-expression of several immune checkpoints, including LAG3, TIGIT, suggesting the promising role of combination of ICIs targeting different immune checkpoints in this group of patients. Given that previous studies have demonstrated that PHLDA family members could mediate resistance to TKI targeted therapy, we investigated the association of expression of PHLDA family members and prognosis of ccRCC patients receiving sunitinib at the same time, whereas no significant difference was observed between subgroups, suggesting different functions in specific types of cancer [16]. Thus, elevated PHLDA2 expression could be served exclusively as a robust biomarker predicting unfavorable outcomes of ICI plus anti-angiogenic agent combination therapy in ccRCC. Further research should be conducted to investigate the predictive role of PHLDA2 for therapeutic efficacy of ICI monotherapy or ICI plus anti-angiogenic agent combination therapy among other cancer types.

Although our study is innovative in investigating the association between PHLDA2 expression and prognosis and treatment responses of ccRCC, it has certain limitations. This study is mainly based on bioinformatics analysis, which needs profound studies to investigate the underlying mechanism of PHLDA2 in the development of ccRCC through in vivo or in vitro experiments. Furthermore, it is still unclear why PHLDA2 expression affects the therapeutic effect of ICI plus anti-angiogenic agent in ccRCC, and further studies are required to explore potential treatment strategies for ccRCC patients with higher PHLDA2 expression.

## Conclusion

Our comprehensive analysis illustrated the expression profile of PHLDA family members in pan-cancer, including ccRCC, for the first time. Further investigation demonstrated that up-regulation of PHLDA2 was associated with adverse clinicopathologic parameters and could be served as an independent high-risk prognostic factor in ccRCC. Elevated PHLDA2 expression was contributed by DNA hypomethylation, and mediated an immunosuppressive microenvironment featured by high infiltrates of Tregs and CAFs. Eventually, using data derived from three RCTs, we confirmed that elevated PHLDA2 expression could robustly predict worse therapeutic efficacy of ICI plus anti-angiogenic agent combination therapy in ccRCC. Our results expand the dimension of precision medicine, and further studies should be conducted to explore the potential role of PHLDA2 as a predictor biomarker for response of ICI or ICI-based combination therapy among other types of cancer.

### Supplementary Information

Supplementary Material 1. Supplementary Fig. 1. Expression pattern and correlations with clinicopathologic parameters and survival of PHLDA1 and PHLDA3 in ccRCC. (A) Expression level of PHLDA1 between tumor and normal tissues in pan-cancer in the TCGA database. *p < 0.05, **p < 0.01, ***p < 0.001. ****p < 0.0001. (B) Expression level of PHLDA3 between tumor and normal tissues in pan-cancer in the TCGA database. (C–H) Correlations between PHLDA1 expression level and clinicopathologic parameters of ccRCC, including age, gender, pT stage, pN stage, metastatic status, ISUP grade. (I–N) Correlations between PHLDA3 expression level and clinicopathologic parameters of ccRCC, including age, gender, pT stage, pN stage, metastatic status, ISUP grade. (O–Q) Associations between PHLDA1 expression and OS, DSS, PFI of ccRCC patients by Kaplan–Meier survival analysis. (R–T) Associations between PHLDA3 expression and OS, DSS, PFI of ccRCC patients by Kaplan–Meier survival analysis.

Supplementary Material 2. Supplementary Fig. 2. Correlations between PHLDA2 expression and mutation status in ccRCC. (A) Expression of PHLDA2 between VHL-wt (wild type) and VHL-mt (mutated type) subgroups in ccRCC. (B) Expression of PHLDA2 between PBRM1-wt and PBRM1-mt subgroups in ccRCC. (C) Expression of PHLDA2 between SETD2-wt and SETD2-mt subgroups in ccRCC. (D) Expression of PHLDA2 between BAP1-wt and BAP1-mt subgroups in ccRCC.

Supplementary Material 3. Supplementary Fig. 3. Correlations between PHLDA2 expression and tumor response of metastatic ccRCC patients receiving immunotherapy, and associations between PHLDA1 and PHLDA3 expression and therapeutic efficacy of systemic treatment in metastatic ccRCC patients. (A) Comparisons of proportion of patients who achieved an objective response between PHLDA2-H and PHLDA2-L subgroups (left), and comparisons of proportion of patients in PHLDA2-L subgroup between noORR (patients who did not achieve an objective response) and ORR (patients who achieved an objective response) subgroups (right) in the nivolumab arm in the CheckMate025 cohort. (B) Comparisons of proportion of patients who achieved disease control between PHLDA2-H and PHLDA2-L subgroups (left), and comparisons of proportion of patients in PHLDA2-L subgroup between noDCR (patients who did not achieve disease control) and DCR (patients who achieved disease control) subgroups (right) in the nivolumab arm in the CheckMate025 cohort. (C) Expression of PHLDA2 between noDCR and DCR subgroups in the nivolumab arm in the CheckMate025 cohort. (D) Comparisons of proportion of patients who achieved disease control between PHLDA2-H and PHLDA2-L subgroups (left), and comparisons of proportion of patients in PHLDA2-L subgroup between noDCR (patients who did not achieve disease control) and DCR (patients who achieved disease control) subgroups (right) in the atezolizumab plus bevacizumab arm in the IMmotion151 cohort. (E, F) Associations between PHLDA1 expression and PFS, OS of metastatic ccRCC patients in the nivolumab arm in the CheckMate025 cohort. (G) Associations between PHLDA1 expression and PFS of metastatic ccRCC patients in the atezolizumab plus bevacizumab arm in the IMmotion151 cohort. (H) Associations between PHLDA1 expression and PFS of metastatic ccRCC patients in the avelumab plus axitinib arm in the JAVELIN101 cohort. (I, J) Associations between PHLDA3 expression and PFS, OS of metastatic ccRCC patients in the nivolumab arm in the CheckMate025 cohort. (K) Associations between PHLDA3 expression and PFS of metastatic ccRCC patients in the atezolizumab plus bevacizumab arm in the IMmotion151 cohort. (L) Associations between PHLDA3 expression and PFS of metastatic ccRCC patients in the avelumab plus axitinib arm in the JAVELIN101 cohort. (M–P) Associations between PHLDA1 and PHLDA3 expression and PFS of metastatic ccRCC patients in the sunitinib arm in the IMmotion151 and JAVELIN101 cohort.

Supplementary Material 4.

## Data Availability

RNA-seq data of treatment-naïve ccRCC primary tumors and adjacent renal tissues collected from West China Hospital, Sichuan University are available from the corresponding author upon reasonable request.

## Abbreviations

ACC: Adrenocortical carcinoma
BLCA: Bladder urothelial carcinoma
BRCA: Breast invasive carcinoma
CESC: Cervical squamous carcinoma and endocervical adenocarcinoma
CHOL: Cholangio carcinoma
COAD: Colon adenocarcinoma
DLBC: Lymphoid neoplasm diffuse large B-cell lymphoma
ESCA: Esophageal carcinoma
GBM: Glioblastoma multiforme
HNSC: Head and neck squamous cell carcinoma
KICH: Kidney chromophobe
KIRC: Kidney renal clear cell carcinoma
KIRP: Kidney renal papillary cell carcinoma
LAML: Acute myeloid leukemia
LGG: Brain lower grade glioma
LIHC: Liver hepatocellular carcinoma
LUAD: Lung adenocarcinoma
LUSC: Lung squamous cell carcinoma
MESO: Mesothelioma
OV: Ovarian serous cystadenocarcinoma
PAAD: Pancreatic adenocarcinoma
PCPG: Pheochromocytoma and paraganglioma
PRAD: Prostate adenocarcinoma
READ: Rectum adenocarcinoma
SARC: Sarcoma
SKCM: Skin cutaneous melanoma
STAD: Stomach adenocarcinoma
TGCT: Testicular germ cell tumors
THCA: Thyroid carcinoma
THYM: Thymoma
UCEC: Uterine corpus endometrial carcinoma
UCS: Uterine carcinosarcoma
UVM: Uveal melanoma
