# Supplementary figures and images for: Epigenetic modification of PHLDA2 is associated with tumor microenvironment and unfavorable outcome of immune checkpoint inhibitor-based therapies in clear cell renal cell carcinoma
Source: Eur J Med Res. 2024 Jul 20;29:378. doi: 10.1186/s40001-024-01939-9 (PMC11264912; doi:10.1186/s40001-024-01939-9)

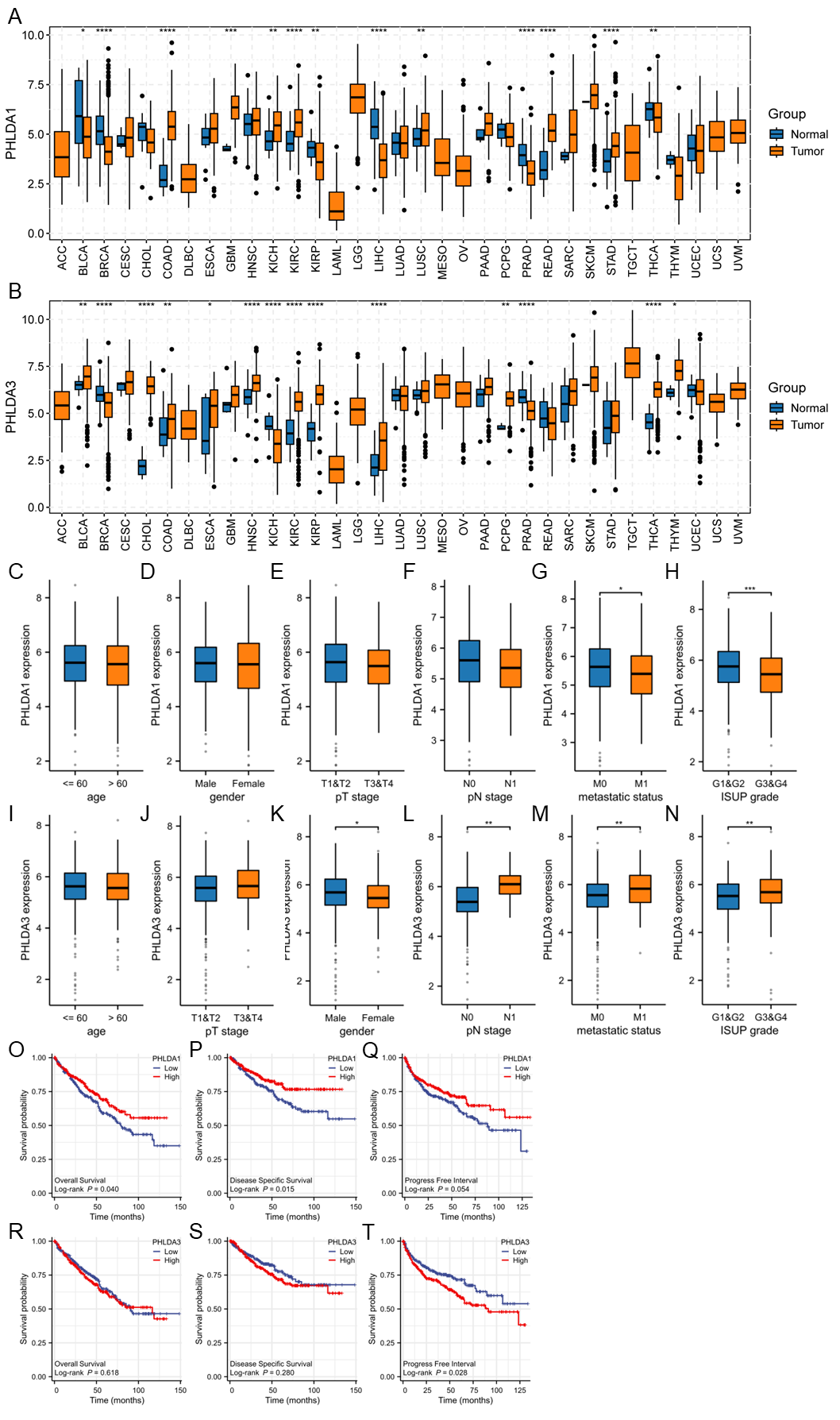

Supplement: Supplementary file 1 — Supplementary Material 1. Supplementary Fig. 1. Expression pattern and correlations with clinicopathologic parameters and survival of PHLDA1 and PHLDA3 in ccRCC. (A) Expression level of PHLDA1 between tumor and normal tissues in pan-cancer in the TCGA database. *p < 0.05, **p < 0.01, ***p < 0.001. ****p < 0.0001. (B) Expression level of PHLDA3 between tumor and normal tissues in pan-cancer in the TCGA database. (C–H) Correlations between PHLDA1 expression level and clinicopathologic parameters of ccRCC, including age, gender, pT stage, pN stage, metastatic status, ISUP grade. (I–N) Correlations between PHLDA3 expression level and clinicopathologic parameters of ccRCC, including age, gender, pT stage, pN stage, metastatic status, ISUP grade. (O–Q) Associations between PHLDA1 expression and OS, DSS, PFI of ccRCC patients by Kaplan–Meier survival analysis. (R–T) Associations between PHLDA3 expression and OS, DSS, PFI of ccRCC patients by Kaplan–Meier survival analysis. [file 40001_2024_1939_MOESM1_ESM.tif]

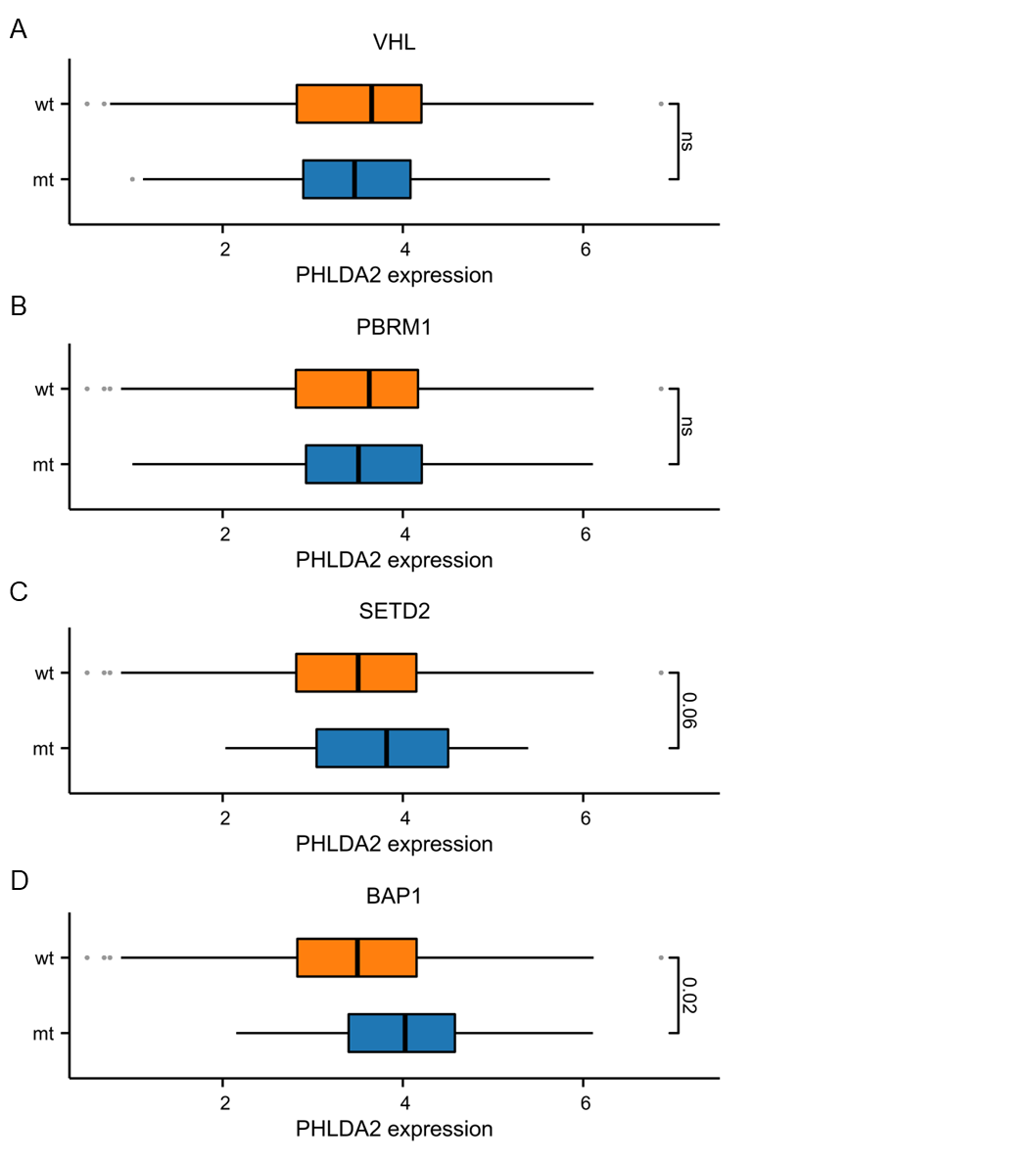

Supplement: Supplementary file 2 — Supplementary Material 2. Supplementary Fig. 2. Correlations between PHLDA2 expression and mutation status in ccRCC. (A) Expression of PHLDA2 between VHL-wt (wild type) and VHL-mt (mutated type) subgroups in ccRCC. (B) Expression of PHLDA2 between PBRM1-wt and PBRM1-mt subgroups in ccRCC. (C) Expression of PHLDA2 between SETD2-wt and SETD2-mt subgroups in ccRCC. (D) Expression of PHLDA2 between BAP1-wt and BAP1-mt subgroups in ccRCC. [file 40001_2024_1939_MOESM2_ESM.tif]

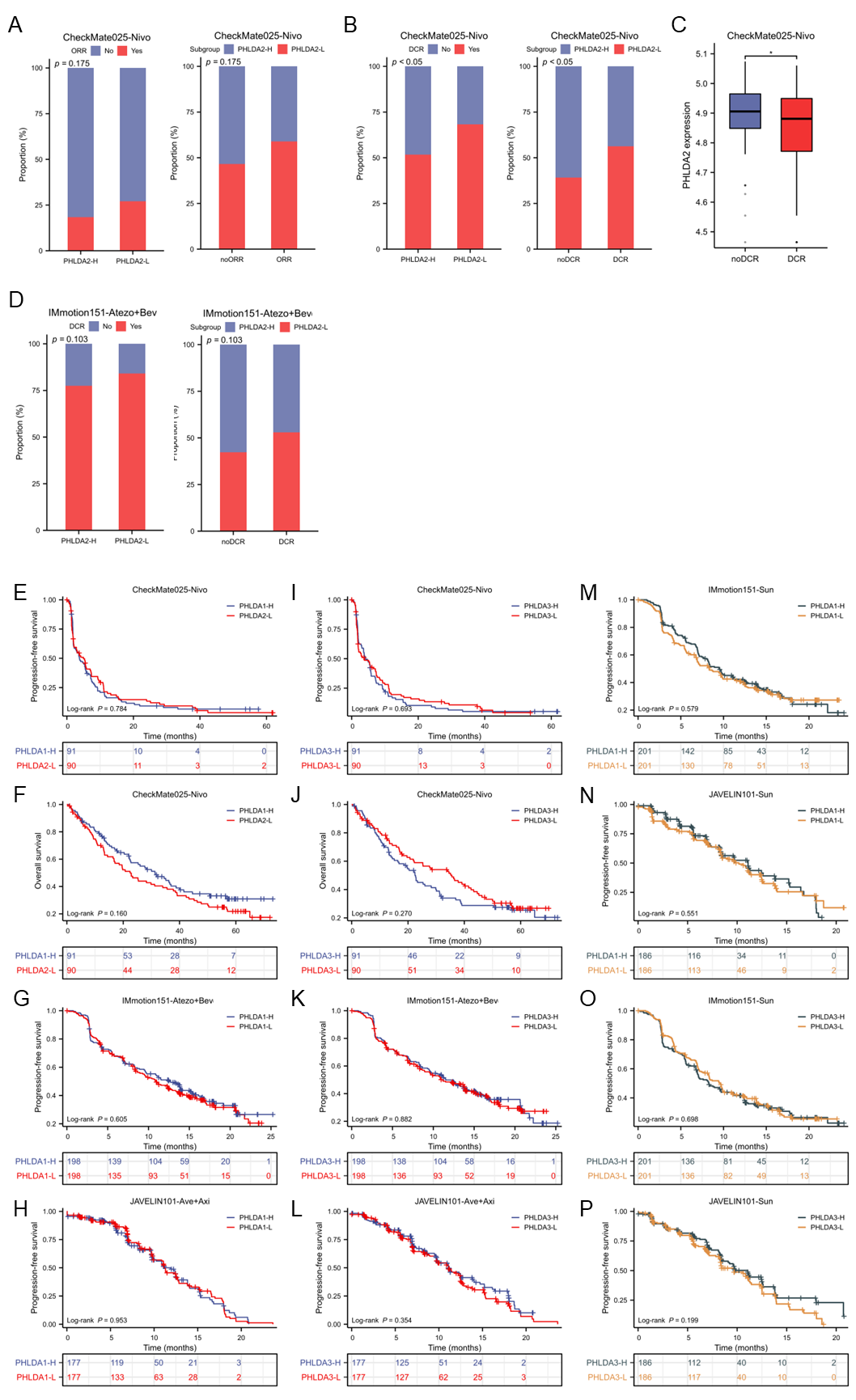

Supplement: Supplementary file 3 — Supplementary Material 3. Supplementary Fig. 3. Correlations between PHLDA2 expression and tumor response of metastatic ccRCC patients receiving immunotherapy, and associations between PHLDA1 and PHLDA3 expression and therapeutic efficacy of systemic treatment in metastatic ccRCC patients. (A) Comparisons of proportion of patients who achieved an objective response between PHLDA2-H and PHLDA2-L subgroups (left), and comparisons of proportion of patients in PHLDA2-L subgroup between noORR (patients who did not achieve an objective response) and ORR (patients who achieved an objective response) subgroups (right) in the nivolumab arm in the CheckMate025 cohort. (B) Comparisons of proportion of patients who achieved disease control between PHLDA2-H and PHLDA2-L subgroups (left), and comparisons of proportion of patients in PHLDA2-L subgroup between noDCR (patients who did not achieve disease control) and DCR (patients who achieved disease control) subgroups (right) in the nivolumab arm in the CheckMate025 cohort. (C) Expression of PHLDA2 between noDCR and DCR subgroups in the nivolumab arm in the CheckMate025 cohort. (D) Comparisons of proportion of patients who achieved disease control between PHLDA2-H and PHLDA2-L subgroups (left), and comparisons of proportion of patients in PHLDA2-L subgroup between noDCR (patients who did not achieve disease control) and DCR (patients who achieved disease control) subgroups (right) in the atezolizumab plus bevacizumab arm in the IMmotion151 cohort. (E, F) Associations between PHLDA1 expression and PFS, OS of metastatic ccRCC patients in the nivolumab arm in the CheckMate025 cohort. (G) Associations between PHLDA1 expression and PFS of metastatic ccRCC patients in the atezolizumab plus bevacizumab arm in the IMmotion151 cohort. (H) Associations between PHLDA1 expression and PFS of metastatic ccRCC patients in the avelumab plus axitinib arm in the JAVELIN101 cohort. (I, J) Associations between PHLDA3 expression and PFS, OS of metasta [file 40001_2024_1939_MOESM3_ESM.tif]
